# Validation of nursing educational technology for neurogenic bowel rehabilitation in people with spinal cord injury

**DOI:** 10.15649/cuidarte.3705

**Published:** 2024-07-09

**Authors:** Geyslane Pereira-Melo-deAlbuquerque, Fabiana Faleiros, Letícia Noelle-Corbo, Luís Sousa, Evanira Rodrigues-Maia, Inácia Sátiro Xavier-de-França, Simone Maria Muniz-da-Silva-Bezerra, Alexsandro Silva-Coura

**Affiliations:** 1 Faculdade de Enfermagem Nossa Senhora das Graças - Universidade de Pernambuco FENSGUPE, Recife (PE), Brasil. E-mail: geyslane.pmalbuquerque@upe.br Universidade de Pernambuco Faculdade de Enfermagem Nossa Senhora das Graças Universidade de Pernambuco FENSGUPE Recife (PE) Brazil geyslane.pmalbuquerque@upe.br; 2 Escola de Enfermagem de Ribeirão Preto/ Universidade de São Paulo (EERP-USP), Ribeirão Preto (SP), Brasil. E-mail: fabifaleiros@eerp.usp.br Universidade de São Paulo Escola de Enfermagem de Ribeirão Preto Universidade de São Paulo Ribeirão Preto Brazil fabifaleiros@eerp.usp.br; 3 Escola de Enfermagem de Ribeirão Preto/ Universidade de São Paulo (EERP/USP), Ribeirão Preto (SP), Brasil. E-mail: leticia.corbo@usp.br Universidade de São Paulo Escola de Enfermagem de Ribeirão Preto Universidade de São Paulo Ribeirão Preto Brazil leticia.corbo@usp.br; 4 Escola de Saúde de Atlântica (ESSATLA), 2730-036 Oeiras, Portugal. E-mail: luismmsousa@gmail.com Escola de Saúde de Atlântica Oeiras Portugal luismmsousa@gmail.com; 5 Universidade Regional do Cariri, Centro de Ciências Biológicas e da Saúde, Crato (CE), Brasil. Email: evanira.maia@urca.br Universidade Regional do Cariri Universidade Regional do Cariri Centro de Ciências Biológicas e da Saúde, Crato Brazil evanira.maia@urca.br; 6 Universidade Estadual da Paraíba UEPB), Campina Grande, PB, Brasil. E-mail: inacia.satiro@gmail.com Universidade Estadual da Paraíba Universidade Estadual da Paraíba Campina Grande Brazil inacia.satiro@gmail.com; 7 Faculdade de Enfermagem Nossa Senhora das Graças - Universidade de Pernambuco FENSGUPE, Recife (PE), Brasil. E-mail: simonemunizm2@gmail.com Universidade de Pernambuco Faculdade de Enfermagem Nossa Senhora das Graças Universidade de Pernambuco Recife Brazil simonemunizm2@gmail.com; 8 Universidade Estadual da Paraíba (UEPB), Campina Grande (PB), Brasil. E-mail: alexcoura@hotmail.com Universidade Estadual da Paraíba Universidade Estadual da Paraíba Campina Grande Brazil alexcoura@hotmail.com

**Keywords:** Nursing, Neurogenic Bowel, People with Disabilities, Rehabilitation, Spinal Cord Injuries, Enfermería, Intestino Neurogénico, Personas con Discapacidad, Rehabilitación, Lesiones de la Médula Espinal, Enfermagem, Intestino Neurogênico, Pessoas com Deficiência, Reabilitação, Traumatismos da Medula Espinal

## Abstract

**Introduction:**

Considered an unpredictable and recurring problem, Neurogenic Bowel does not resolve over time and progressively worsens, translating into a physical and psychological challenge, significantly reducing Quality of Life.

**Objective:**

To construct and validate the face and content of an educational technology for use by nurses in the rehabilitation of Neurogenic Bowel in people with Spinal Cord Injury.

**Materials and methods:**

A methodological, quantitative study developed in two stages: the construction of an educational technology on the Canvas platform based on a literature review based on Wanda Horta's Theory of Human Needs and its validation by expert judges. The validation process included nurses with ability in neurogenic bowel in teaching, research or care. The criterion for validation was agreement of over 80%, analyzed using the Content Validation Index and binomial test with confidence intervals of 95.00% (p<0.05).

**Results:**

The integrative literature review proved necessary for the construction of the proposed educational technology and covered characteristics of the Neurogenic Bowel, bowel emptying techniques, guidelines and the systematization of nursing care. The protocol was validated by ten expert judges who had graduated in nursing for more than 10 years (100.00%), with an average age of 41.8 years, female (60.00%) and an average training period of 18.6 years. An overall Content Validation Index of 0.96 (p<0.001) was obtained for the items assessed (objective, content, relevance, functionality, efficiency and appearance/diagramming).

**Discussion:**

The implementation of an intestinal rehabilitation program aims, above all, to achieve frequent, regular and consistent stools in people with spinal cord injury and nurses, as multipliers of knowledge, can be facilitators in the teaching-learning process for carers, people with this condition and other members of the healthcare team.

**Conclusion:**

It can be concluded that the educational technology has been validated and could help the teaching-learning process for nurses in the clinical practice of caring for people with Neurogenic Bowel Disease.

## Resumen

**Introducción:**

Considerado un problema impredecible y recurrente, el Intestino Neurogénico no se resuelve por sí solo con el tiempo y se vuelve progresivamente más pronunciado, traduciéndose en un desafío físico y psicológico, reduciendo significativamente la Calidad de Vida.

**Objetivo:**

Construir y validar el rostro y el contenido de una tecnología educativa para uso del enfermero en la rehabilitación del Intestino Neurogénico en personas con Lesión Medular.

**Materiales y Métodos:**

Estudio metodológico, cuantitativo, desarrollado en dos etapas: construcción de una tecnología educativa en la plataforma Canvas a partir de una revisión de literatura basada en la Teoría de las Necesidades Humanas de Wanda Horta y validación por jueces expertos. Para la validación se incluyeron enfermeros con experiencia en Intestino Neurogénico en la docencia, investigación o asistencia. El criterio de validación fue la concordancia mayor al 80%, analizado mediante el Índice de Validación de Contenido y prueba binomial con intervalos de confianza al 95,00% (p<0,05).

**Resultados:**

La revisión integradora de la literatura resultó necesaria para la construcción de la tecnología educativa propuesta e incluyó características del Intestino Neurogénico, técnicas de vaciamiento intestinal, guías y la sistematización de los cuidados de enfermería. Diezjueces expertos graduados en enfermería hacen más de 10 años (100,00%), con edad promedio de 41,8 años, predominantemente del sexo femenino (60,00%) y tiempo promedio de formación de 18,6 años, validaron el protocolo. Se obtuvo un Índice de Validación de Contenido general de 0,96 (p<0,001) en los ítems evaluados (objetivo, contenido, relevancia, funcionalidad, eficiencia y apariencia/diseño).

**Discusión:**

La implementación de un programa de rehabilitación intestinal tiene como objetivo, sobre todo, obtener deposiciones frecuentes, regulares y consistentes en personas con Lesión Medular y el enfermero como multiplicador de conocimientos puede ser un facilitador en el proceso de enseñanza- aprendizaje de los cuidadores, de las personas. con esta afección y para otros miembros del equipo de atención médica.

**Conclusión:**

Se concluye que la tecnología educativa está validada y puede facilitar el proceso de enseñanza-aprendizaje del enfermero en la práctica clínica del cuidado de personas con Intestino Neurogénico.

## Resumo

**Introdução:**

Considerado um problema imprevisível e recorrente, o Intestino Neurogênico não se resolve com o tempo e se acentua progressivamente, traduzindo-se em um desafio de caráter físico e psicológico, diminuindo significativamente a Qualidade de Vida.

**Objetivo:**

Construir e validar face e conteúdo de uma tecnologia educativa para uso de enfermeiros na reabilitação do Intestino Neurogênico em pessoas com Lesão Medular.

**Materiais e métodos:**

Estudo metodológico, quantitativo, desenvolvido em duas etapas: construção de uma tecnologia educativa na plataforma Canvas a partir da revisão da literatura fundamentado na Teoria das Necessidades Humanas de Wanda Horta e validação dele por juízes experts. Para a validação foram incluídos enfermeiros com expertise em Intestino Neurogênico na docência, pesquisa ou assistência. O critério para validação foi concordância superior a 80%, analisada por meio do Índice de Validação de Conteúdo e teste binomial com intervalos de confiança em 95,00% (p<0,05).

**Resultados:**

A revisão integrativa da literatura mostrou-se necessária para a construção da tecnologia educativa proposta e contemplou características do Intestino Neurogênico, técnicas de esvaziamento intestinal, orientações e a sistematização da assistência de enfermagem. Validaram o protocolo dez juízes experts graduados em enfermagem há mais de 10 anos (100,00%), com idade média de 41,8 anos, predominantemente do sexo feminino (60,00%) e tempo médio de formação de 18,6 anos. Obteve-se Índice de Validação de Conteúdo geral de 0,96 (p<0,001) nos itens avaliados (objetivo, conteúdo, relevância, funcionalidade, eficiência e aparência/diagramação).

**Discussão:**

A implementação de um programa de reabilitação intestinal objetiva, sobretudo, a obtenção fezes frequentes, regulares e consistentes em pessoas com Lesão Medular e o enfermeiro enquanto multiplicador de conhecimento pode ser um facilitador no processo de ensino aprendizagem para os cuidadores, para as pessoas com esta condição e para outros membros da equipe de saúde.

**Conclusão:**

Conclui-se que a tecnologia educativa está validada e poderá facilitar o processo de ensino-aprendizagem do enfermeiro na prática clínica de cuidado com pessoas com Intestino Neurogênico.

## Introduction

Neurogenic Intestine (NI), understood as an important neurological damage compromising intestinal elimination, is considered one of the main risk factors for morbidity and mortality in people with Spinal Cord Injury (SCI). Individuals with this condition present interruption of nervous stimuli regarding the need for evacuation, negatively affecting quality of life^1^. Among the main causes of IN, around 70.00 % to 80.00 % can be attributed to sequelae of Spinal Cord Injury (SCI)^2^. In the face of intestinal involvement secondary to SCI, fecal incontinence occurs in 78.00 % of spinal cord injuries and constipation in 38.00 %^3^.

The pattern of intestinal irregularity after SCI are prerequisites for high morbidity, as observed in a cross-sectional study carried out in the city of Ribeirão Preto (São Paulo) with 22 individuals, whose results indicate intestinal constipation (68.20%) and total dependence for use of the toilet (36.40%)^4^. Research carried out in Denmark identified a prevalence of 35.00 % of fecal incontinence and 79.00 % of intestinal dysfunction in 684 women with SCI. Depending on this, additional problems were added, such as urinary incontinence, decreased quality and satisfaction of general and psychological life^5^.

Configured as an unpredictable and recurring problem, IN is not resolved over time and becomes more pronounced with advancing age, translating into a physical and psychological challenge, significantly reducing Quality of Life (QoL). The clinical impact is reflected in the percentage of approximately 11.00 % of hospitalizations after SCI and in side effects including fecal impaction, mega colon, rectal bleeding, prolapse, formation of anal fissures, chronic constipation and fecal incontinence^6^.

Considering the need for support, comprehensive and humanized health care for people with NI and other disabilities, Brazil has implemented public policies, laws and plans to support the exercise of legal capacity by these individuals. Determined by Ordinance No. 1,526, of October 11, 2023, the National Policy for Comprehensive Health Care for People with Disabilities and the Care Network for People with Disabilities (RCPD) establishes the inclusion of individuals with disabilities within the scope of the System National Health System (SUS), considering the needs of these subjects in all their dimensions^7^^, ^^8^.

On the other hand, the National Plan for the Rights of Persons with Disabilities, implemented by Decree No. 7,612, of November 17, 2011, articulates between the federal, state and municipal spheres the access of these people to education, social inclusion, health care and accessibility^9^. As legal support we have Law No. 13,146 of 2015, entitled Brazilian Law for the Inclusion of Persons with Disabilities, which emphasizes the right to accessibility, inclusive education, rehabilitation, biopsychosocial assessment and inclusion assistance as a stimulus for entry into the market. Work^10^.

Therefore, the individual with IN must be inserted into an intestinal rehabilitation program as soon as the cause is stabilized, and it must comprise a fundamental approach of nurses from the preparation for hospital discharge in Urgency and Emergency, involving the person, family members and home caregivers. Preparation that must be extended in services and centers specialized in rehabilitation and reiterated in Primary Health Care as short, medium, and long-term care. Therefore, the earlier the care and appropriate management, the better the results when faced with this problem, reducing impacts on the outcomes of clinical manifestations, in addition to minimizing complications, which can be fata^3^^, ^^5^^, ^^6^.

When considering educational technologies (ET) as a management instrument, organization of health work and guidance of care, nursing protocols are seen as tools for promoting teaching- learning constructed and validated by experts who use guidelines and scientific evidence to guide care practices^11^. Additionally, a randomized clinical study carried out in China with 50 people with IN after SCI identified that after implementing a nursing intestinal rehabilitation program, patients in the intervention group showed better recovery of intestinal function, greater quality of life and satisfaction with life, when compared to the control group who received routine guidance and health education^12^.

In Brazil and other countries, there are studies that recommend the development of technologies that enable clinical assessment and systematized care for people with NI in order to offer care that improves the quality of life and encourages the autonomy of these people^1^^, ^^13^. In this scenario, nursing care implemented with the help of systematized protocols can allow better adherence, understanding, identification of problems and guidance of conduct in a safe and quality manner. Additionally, TE, such as hypermedia, protocols and booklets validated for the Brazilian public, are not always available, making it impossible to democratize information for people from various Brazilian locations^14^.

Accordingly, nursing theories are presented as methodological bases that offer support for care work based on patients' needs^15^. Understanding the biopsychosocial needs of patients with NI, Wanda de Aguiar Horta's Theory of Basic Human Needs was chosen to support the present study, as it is a theory that guides nursing care in the basic needs of individuals.

According to Horta's theory, the nurse's assistance must be carried out in a planned and resolute manner through a satisfactory analysis of the patient's general condition, seeking a balance between their well-being in time and space. Carried out in a systematized and interrelated way, health actions need to offer the resolution of problems presented by individuals in terms of prevention, recovery and rehabilitation at all stages of life^16^.

In addition, a methodological study, when creating and validating the process of developing a hospital protocol for nursing care for patients with intestinal stomas, considered the material produced as appropriate and useful for nursing care for hospitalized patients with a stoma in the evaluation of stomatologists and nurses. Assistance^17^.

In this way, understanding the development of technologies, innovation, and evaluation of the implementation of health strategies in the SUS as priorities in the National Agenda of Health Research Priorities, it is understood that all technical skills are fundamental for nurses. However, the main skills that make you capable of solving complex scenarios require critical reasoning and the ability to lead your team^18^.

In view of the above, the present study aimed to build and validate the face and content of an educational technology for use by nurses in IN rehabilitation.

## Materials and Methods

### Study design, location and data collection period

Methodological study with a quantitative approach developed in two sequential stages. The first stage dealt with the construction of the protocol and the second with its validation by *expert judges*. The study was carried out in a virtual environment and data collection spanned the period from October 2021 to February 2022. The data set was stored in Zenodo^19^.

### Sample and inclusion and exclusion criteria

To recruit participants, electronic contacts were obtained from the Lattes Platform of nurses with experience in intestinal rehabilitation using the *snowball technique*, to integrate the sample at this stage of the study. The inclusion criteria were nurses with experience in IN rehabilitation with at least one year of experience in teaching, research and/or clinical practice. Failure to complete the data collection instrument was considered as an exclusion criterion. In total, 22 invitations were sent, however only 10 *expert judges* agreed to participate in the study.

To enable the achievement of the study objective, it was necessary to adapt the data collection instrument^20^, consisting of 27 items containing *Likert* Scale responses, in which values of 1 (Inadequate), 2 (Poorly Adequate) were assigned to each criterion.), 3 (Quite Adequate) and 4 (Very adequate - no need for correction). The first ten who sent the fully completed form were included in the sample.

### Protocol Construction

Survey of the Theoretical Framework: To enable the operationalization of the research, it was necessary to deepen knowledge and synthesize the results of research on nursing care for IN rehabilitation. After critical reading of the publications available in the literature, 16 scientific articles, two books and a technical manual from the Brazilian Ministry of Health (MS) were selected.

Technology was mapped in light of Wanda Horta's Theory of Basic Human Needs^21^, considering the following assumptions: Psychosocial, Psychoemotional and Psychobiological Needs with a focus on the teaching-learning process to promote self-care in its functional implications specific to the person with LM and rehabilitation of IN through conservative maneuvers that aid intestinal emptying.

Planning: The content was organized containing “branched” topics to allow the reader to choose the study points of greatest interest. Data from similar subjects were analyzed and brought together to capture all possible important information.

Text insertion: After organizing the content, the textual production was prepared with the aim of focusing on describing criteria used in the step-by-step process and the care provided in the rehabilitation of the IN. The texts produced underwent vernacular correction, aiming to avoid grammatical errors and deviations from the Portuguese language.

Diagramming and Layout: the technology was formatted following the recommendations of Standards No. 6029 of the Brazilian Association of Technical Standards (ABNT) and organized in a way that included the External Part, containing Cover, Back Cover and Internal Part, subdivided into: (i) Pre -Textual Elements, which include title page, authors, titles and qualifications, title, location, year and acknowledgments; ( ii ) Textual Elements, composed of presentation and content; and ( iii ) Post Textual Elements: which contains references^22^.

Both the text and the illustrations were implemented on the *Canvas virtual platform, a web -accessible* management tool that enables the development of applications, solutions, educational programs, *folders*, booklets, protocols and educational materials.

### Validation of Educational Technology

The Educational Technology was validated according to the objective (referring to the purposes, goals and desirable ends using the protocol); content (content, topics, including general organization, structure and presentation strategy), relevance (characteristics that assessed the degree of significance); functionality (functions and properties to assist nurses’ learning process); efficiency (ability to present performance for learning) and appearance/layout ( ability to evaluate appearance and organization.

### Analysis of results and statistics

In the validation analysis, the prevalence of adequate judgment by the judges was obtained for the items of the instrument from which the Content Validity Index (CVI) was calculated and the Binomial Test was applied to evaluate statistical similarity with the minimum score of 0.80. All conclusions were drawn considering a significance level of 5%^23^. In this way, the TE was validated after 80% of the judges assigned the concept of 3 (very adequate) and/or 4 (adequate without need for correction) in each item evaluated, not requiring a new round, analysis and reformulation of the items.

The collected data were transferred and analyzed using the statistical program *Statistical Package for the Social Sciences®* - *SPSS base for Windows*® 20.0 and the *Microsoft Office Excel*® 2010 program. For the descriptive analysis, the frequency and percentage calculation were carried out, the mean and standard deviation were calculated considering the confidence intervals in 95.00% (p<0.05).

### Ethical aspects

The project was approved by the Research Ethics Committee (CEP) under Opinion No. 4,643,887 and obtained co-participation approval from the CEP of a second institution, under Opinion No. 4,768,605, according to Resolution 466/12, of the National Council of Research Ethics of the Ministry of Health, which addresses ethics in research with human beings.

## Results

### Construction of the IN rehabilitation protocol

Based on the results obtained through the literature, nursing care for people with NI was based on scientific evidence, to enable the planning of intestinal rehabilitation to offer greater autonomy, participation and QoL. Initially, the theme, content, target audience and application objectives were structured in accordance with the ethical and legal precepts of the Federal Nursing Council (COFEN)^24^. Therefore, it was presented as a constructivist proposal for the teaching-learning process containing cover, summary, presentation of the theme and content organized and distributed didactically in 45 pages. With the purpose of widely disseminating the ET, it was published on the D+ Information Portal available at the link: https://demaisinformacao.com.br/protocolo-de-reabilitacao-do-intestino- neurogenico-para-enfermeiros/

Aiming for a playful and inviting organization, TE outlined the script for the general and specific physical examination, external and internal rectal examination, positioning and maneuvers for manual removal of feces. To strengthen the health education process, a reminder of intestinal function and illustrations with guidelines focusing on a diet rich in fiber and liquids were included, to enable communication between the nurse, caregiver and/or person with IN.

**Figure 1:**
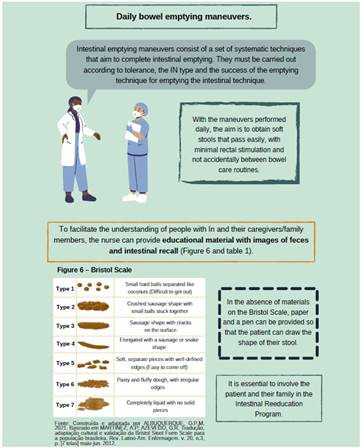
Images of educational technology

**Figure 2:**
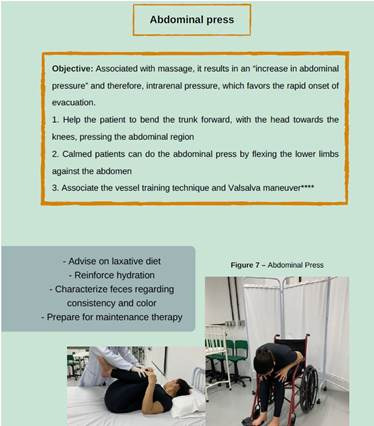
Images of educational technology

**Figure 3:**
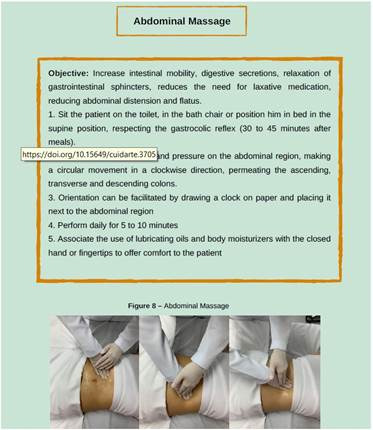
Images of educational technology

**Figure 4:**
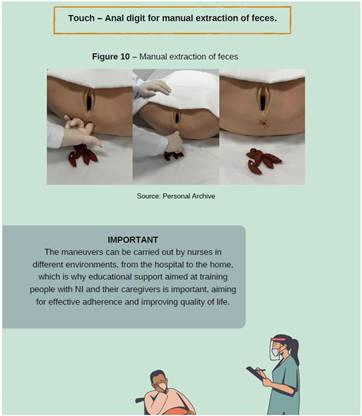
Images of educational technology

About conservative maneuvers for IN rehabilitation, it was pointed out that this is a strategy that brings together several actions aimed at promoting the autonomy of individuals, preventing complications and facilitating intestinal elimination, as shown in Figures 1, 2, 3 and 4.

*Personal collection, 2023*

*Personal collection, 2023*

*Personal collection, 2023*

*Personal collection, 2023*

At the end of the ET, a nursing care plan was listed based on the following Nursing Diagnoses according to the NANDA Taxonomy^25^: Constipation, Fecal incontinence, Pain, Deficit in self-care for feeding, Risk of autonomic dysreflexia , Risk of infection, Risk of impaired skin integrity, intestinal incontinence and self-care deficit for intimate hygiene.

### Validation of the IN-rehabilitation protocol

Regarding the validation of the protocol developed in this study, it is noted that the TE obtained a significant number of *expert judges* (10), following the recommendations of previous research^26^, which provided an analysis of the construct from the perspective of professionals in the field academic and assistance. Regarding the number of experts to compose a validation of teaching material, there are controversies in the literature. Authors state that the quantity must be composed of at least five and at most ten^27^, six to twenty subjects^28^, or a minimum number of six^29^. Based on the diversity of opinions, all stages of this study had a minimum of six^29^ and a maximum of ten judges^27^.

Table 1 shows the distribution of participant characteristics. It was observed that 60.00% (6) were female, 100.00% (10) were over 30 years old, 70.00% (7) were married and all (10) had completed more than 10 years or more. They had an average age of 41.8 years (SD= 6.0) and an average training time of 18.6 years (SD= 6.1).

**Table 1:** Distribution of the personal profile and training of expert judges, Recife, PE, Brazil, 2023

Factor evaluated	N (n=10)	%
Gender		
Female	6	60.00
Male	4	40.00
Age		
Over 30 years old	10	100.00
Mean ± Standard deviation	41.80±6.00	
Marital Status		
Single	3	30.00
Married	7	70.00
Training time		
10 years or more	10	100.00
Mean ± Standard deviation	18.60±6.10	

Table 2 shows the prevalence of *expert judges' experiences.* The most cited were: 90.00% (9) with clinical practice of at least 5 years in intestinal rehabilitation and/or neurogenic intestine, 60.00% (6) doctors, 50.00% (5) with participation in scientific event in the last two years on intestinal rehabilitation and/or neurogenic intestine and 40.00% (4) with publication in an indexed journal on intestinal rehabilitation and/or neurogenic intestine. It is important to infer that the expert judges presented a Fehring^(30)^ score higher than 8 in the present study.

**Table 2:** Prevalence of the experiences of expert judges, Recife, PE, Brazil, 2023

Evaluated factor	n	Prevalence (n=10)
Clinical practice in the area of interest for at least 5 years	9	90.00
Doctor 's degree	6	60.00
Participation in a scientific event in the area of interest in the last two years	5	50.00
Publication in an indexed journal in the area of interest	4	40.00
Master's title	3	30.00
Specialization in the area of interest	1	10.00
Guidance on academic work in the area of interest	1	10.0
Participation in work evaluation boards in the area of interest	1	10.0

*Area of interest: neurogenic bowel rehabilitation

Table 3 shows the CVI values of the items evaluated in the ET domains. At this stage, objective and relevance items were highlighted with 100% agreement. It was found that all items evaluated presented a CVI greater than or equal to the minimum reference value of 80%^27^.

**Table 3:** CVI analysis of items related to the validation of educational technology, Recife, PE, Brazil, 2023. (n=10)

Domain / evaluated item	^to^ IVC	^b^ p- value
Protocol objectives		
The goals they are clear	1.00	0.107
The chosen verbs are precise	1.00	0.107
Are consistent with the content presented	1.00	0.107
IVC domain	1.00	0.001
Protocol content		
Meets the theme and objectives proposed for the rehabilitation of the Neurogenic intestine	1.00	0.107
It is updated and contains correct information	1.00	0.107
Texts are easy to read	1.00	0.107
The writing style is compatible with the level of the proposed target audience	1.00	0.107
The information is well structured in terms of agreement and spelling	0.90	0.376
The content of proposed Neurogenic Bowel rehabilitation care is based on scientific evidence	1.00	0.107
It is consistent with the reality of care environments for patients with Neurogenic bowel	1.00	0.107
Topic content follows a logical sequence	0.90	0.376
The content allows the understanding of the topic	1.00	0.107
IVC domain	0.98	<0.001
Relevance of the protocol		
The protocol contributes to learning and knowledge acquisition	1.00	0.107
Addresses necessary topics for nurses in a safe way	1.00	0.107
It is suitable for use by nurses	1.00	0.107
IVC domain	1.00	0.001
Protocol functionality		
The protocol can be used as a teaching resource efficiently	1.00	0.107
Support functions are well implemented	0.90	0.376
Presents easy mechanisms for learning about neurogenic intestine	1.00	0.107
IVC domain	0.97	0.011
Protocol efficiency		
The proposed number of pages is compatible with the amount of content	0.90	0.376
Resources are used appropriately and understandably	0.80	0.624
IVC domain	0.85	0.411
Protocol appearance / layout		
Information on the cover, back cover, acknowledgments and/or presentations is coherent	1.00	0.107
The title size and topics are adequate	1.00	0.107
The textual elements comply with what is proposed for the protocol	0.90	0.376
Images are relevant to the information included in the text	0.90	0.376
The size and font type of the content are appropriate	0.90	0.376
The amount of information inserted on each page is adequate	1.00	0.107
The choice of font colors in the protocol is adequate	0.80	0.624
IVC domain	0.93	0.003
IVC- general	0.96	<0.001

alVC - Content Validity Index bp -value ofthe Binomial test H 0 : IVC = 0.80 x H 1 : IVC * 0.80.

The binomial test was non-significant in all factors evaluated (p-value greater than 0.05), indicating that all domains showed a similar level of agreement between the judges, confirming the validation of the TE. In the evaluation by domain, all domains presented CVI statistically higher than the reference value, except for the protocol efficiency domain in which p- 0.411, indicating that the CVI value = 0.85 are statistically like the reference value 80.00%. Furthermore, the results showed that the ET was considered understandable and validated, with no need for a new round of protocol analysis by the judges.

Two judges suggested modifying the tone of the background and flowcharts to a softer color, three judges suggested removing the images of fruits that existed in the diet topic, justifying that they did not add knowledge (fruits), thus it was decided to accept the considerations.

In addition to this aspect, it was considered that, as it is a teaching material containing information that covers everything from the legislation of the nursing profession to the SAE, the content could be read at different times if the reader wishes and/or is available. From the suggested comments, the validity of the analyzed items is reinforced.

## Discussion

This study derived a technological innovation aimed at nurses for the rehabilitation of people with NI. Accordingly, the literature highlights the educational component as part of the clinical management strategies and identifies the nurse as the protagonist for the multiplication process regarding anatomy, defecation process, effect of SCI on intestinal function and the description of the intestinal program in a playful and accessible^1^^, ^^2^^, ^^13^^, ^^17^. It is important to highlight that, in addition to bowel emptying maneuvers, other important information that was part of the protocol content highlighted the definition, causes, types of IN and nursing diagnoses.

In this sense, research carried out in London revealed the feeling of dissatisfaction in performing intestinal care, on the part of nurses, due to the difficulties in systematizing the techniques adopted and the need for training for the entire nursing team^31^. The implementation of an intestinal rehabilitation program aims, above all, to obtain frequent, regular and consistent stools according to the experiences of a research group from the Ribeirão Preto School of Nursing at USP, which identifies as the main maneuvers: fecal disimpaction ; massage and abdominal press; vessel training associated with the Valsalva maneuver ; the use of enemas ; and a diet rich in fiber with water intake and physical activity can be considered^32^. Accordingly, people with NI present a series of difficulties inherent to this condition that contribute to the interruption of rehabilitation programs, such as excessive repetitive effort of the upper limbs, which are essential for the movement of these people to perform NI management and in the therapeutic itinerary of your home to health services. These facts confirm the relevance of the ET developed in this study, which included flowcharts and images detailing care techniques for intestinal emptying.

The development and validation of the protocol for the rehabilitation of IN corroborates the inclusion and accessibility recommended by Law 1346/2015, which provides for the Inclusion of People with Disabilities with regard to safety and promotion, under conditions of equality, the exercise of rights and fundamental freedoms for people with disabilities, aiming at their social inclusion and citizenship, as well as with Ordinance 793 of 2012 and 1,526 of 2023^7^^, ^^8^^, ^^24^.

Accordingly, it corroborates the information released in the last Brazilian census about 23.9% of its population having some type of disability, and of these 11.3% correspond to motor disabilities^32^^, ^^33^. That said, it was observed that the stage of construction of the TE on the rehabilitation of the IN, through rigorous investigation of the scientific literature in order to identify the demands of this segment of the population on the subject addressed, represented essential phases for planning the topics and the line of care so that nurses can best meet the needs of these people, using an accessible, clear and understandable protocol.

In this context, the literature highlights the educational component as part of the clinical management strategies for IN. To this end, the nurse, as a multiplier of health education, must provide knowledge about anatomy, the defecation process, the effect of LMT on intestinal function and the description of the intestinal program in a playful and accessible way^11^^, ^^31^^, ^^33^. It is important to highlight that, in addition to bowel emptying maneuvers, other important information that was part of the protocol content was about the definition, causes, types of IN and complementary activities, such as diet and physical exercise.

Accordingly, there is a growing concern in the nursing field about the need to develop methodologically rigorous research that offers security for care practice. In this way, convergent care research has been instituted to give a new meaning to nursing studies, uniting knowing-thinking with knowing-how. In this sense, nursing protocols have the ability to recommend nursing actions and care in a systematized manner and based on scientific evidence, in order to promote greater quality and guaranteed health care^34^.

In this sense, a methodological study produced and validated an educational video on intestinal emptying maneuvers to train individuals with IN in the intestinal rehabilitation process, revealing the nurse as a preponderant actor in preparing the individual for intestinal rehabilitation^32^. Therefore, the role of the nurse as a multiplier of knowledge is highlighted as fundamental to the process of adherence and success of intestinal rehabilitation. The actions begin from the pathophysiology of IN, reaching the management options for fecal elimination, transcending beyond the physical body, considered an object of care^12^^, ^^13^. Furthermore, the nurse is an agent of change in a perverse reality in the daily lives of these people, many without access to rehabilitation programs and with several doubts about how to safely carry out their daily activities.

Systematized maneuvers, also called conservative, can be recommended by nurses from basic to specialized care, having a low cost and little risk of negligence, malpractice and imprudence, favoring safe assistance with a reduction in interventions. There are many factors that influence the results of the studies analyzed, namely: the association of maneuvers with greater chances of positive results, health education with patients and families, patient acceptance and adaptation to the guidelines.

Regarding the validation of the ET, a significant number of judges were scored capable of providing an analysis of the construct from the perspective of professionals from the academic and care sectors. It is important to highlight that most participants were female, corroborating other ET validation studies in nursing^7^^, ^^21^^, ^^32^ reaffirming the still persistent feminization in the profession.

The predominance of masters and doctors with experience in clinical practice on NI and rehabilitation consolidated the literature guidelines on the importance of specialists having, in addition to their title, professional knowledge about the topic covered in the material under validation process^35^. In this way, the possibility of selecting judges working in the triad of teaching, research and assistance was considered, as evidenced by a Brazilian study that developed and validated a TE, to increase the health benefits of the population^7^.

It is worth mentioning that the CVI of all domains evaluated presented a score higher than 80.00%, suggesting that the intestinal rehabilitation protocol was considered validated. Studies developed on ET validation also presented CVI similar to the results of this research^36^^, ^^37^. The same understanding was perceived by authors who constructed and validated ET for the prevention of complications in intestinal ostomies and periostomy skin that presented CVI above 80.00% in all items^37^.

Regarding the analysis of the Objective item, research carried out with 27 experts when constructing and validating the Health Educational Content Validation Instrument (IVCE) identified the importance of the “objective” domain in educational technology assessment instruments to provide greater understanding of the content of material^37^. Educational objectives can be characterized by the scope of learning that certain educational material will achieve in its target audience. Furthermore, they guide the reader about what awaits them during the reading, it is the moment when they define what they are going to learn, making the practice easier and more enjoyable^12^^, ^^18^.

Regarding the content of the protocol, the judges considered the information clear and easy to understand. Similarly, such agreements were also found by other researchers, who highlighted that when preparing educational materials, technical-scientific information must present clarity and simplicity compatible with the target audience^38^.

*Expert judges* assessed the relevance of the TE developed, considering it adequate. The material addresses important aspects for nursing care in NI care and is in accordance with the profession's legislation, in addition, it intensifies learning and the acquisition of knowledge through updated sources.

It is also evident that technology can help reduce the complications inherent to IN, since intestinal emptying strategies with conservative techniques allow self-care, the inclusion of these people in social, sexual and work scenarios, making it essential to disseminate the material among nurses so that they can use and, consequently, understand the aspects covered in order to support them in making safe decisions^33^.

The results from the *expert judges* revealed that the ET developed uses teaching-learning resources efficiently, with interaction mechanisms between the nurse and the patient as it offers the possibility of dialogue before, during and after intestinal rehabilitation maneuvers, communication and support functions (nursing practice legislation, history of NHB theory and clinical management guide) for safe and quality care.

Nurse Wanda Horta developed the Theory of Human Motivation, also known as the Theory of Basic Human Needs based on the context of Brazilian Nursing, considering fundamental aspects such as: laws of balance (homeostasis), adaptation and holism, in addition to focusing attention on NHB demonstrations. For Horta, such needs are flexible, cyclical, interrelated and present in the lives of all people^39^.

The NHB theory was then considered as a guide to elucidate nursing care for individuals with IN. The selection of this theory was motivated by its suitability for the initial phase of this intestinal condition, in which people commonly depend directly on the help of professionals and family members to carry out their intestinal elimination satisfactorily.

It should be noted that the NHB of people with NI undergo changes, as this complication can result in disorders of nutrition, sleep and rest, sexuality, safety, freedom, leisure, self-esteem, independence and intestinal elimination. In this way, the nurse becomes an indispensable protagonist in the reconstruction of physical, emotional and biological balance, promoting nursing care that ranges from bowel emptying, to the promotion of autonomy and better QoL^40^^, ^^41^.

Regarding validation of the appearance and layout of the protocol, the *expert judges* showed positive percentage agreement, corroborating studies carried out in the country^42^^, ^^43^. However, there was a polarization in the different suggestions regarding the size of the material and the background colors used on the pages, tables, tables and flowcharts. It was possible to verify that the judgments about color were not accompanied by suggestions that would make other tone and color options viable.

Regarding suggestions, it is important to note that at the end of each item there was space to indicate suggestions and make comments, pertinent corrections and recommendations for improving the protocol. The spaces available at the end of the instruments so that the interviewee can make notes such as questions and suggestions is important as it allows communication between study participants and researchers^44^.

It is mentioned by authors that TEs must be pleasant to guide and capture attention. In this sense, images and figures must offer the opportunity to represent the concepts of the content in a similar way^45^. Other studies used image resources, flowcharts and insertion of *links* in the construction of educational technologies with the aim of deepening the teaching-learning process on the topic^46^. When considering the nurses' routine and the great demand for activities, the insertion of figures and flowcharts in the technology is relevant so that the information becomes more accessible and targeted.

Currently, there are a growing number of health services with internet access with computers or cell phones. This aspect highlights the relevance of making the technology available in a virtual manner in the future, expanding the information available and reaching various national and international segments.

Finally, it is worth highlighting that the main limitation of this study concerns the non-intervention of applicability in rehabilitation centers so that nurses working in care practice could evaluate its effectiveness. Another limitation refers to the validation stage of the data collection instrument due to the difficulty in obtaining answers. It is believed, however, that such limitations did not negatively influence the validity of this study, however, it provides knowledge for future research in the area.

## Conclusion

Nursing care protocols have emerged, in recent years, as important educational technologies in the teaching-learning process. Both the verification of the care offered by nurses and the maneuvers for clinical management of IN found in the literature were essential for identifying the gaps and main demands of the target audience regarding intestinal rehabilitation needs. In this way, it corroborated with better guidance on the development of the content of the care protocol.

The protocol developed represents a potentially significant resource for promoting teaching-learning, focused on nursing care in accordance with the precepts announced by Wanda Horta in her Theory of Basic Human Needs. In this sense, considering the information and image/mental model inserted in the protocol, nurses will be able to learn the new information so that they adopt safe practices and attitudes in their daily lives with a view to the rehabilitation of IN.

The protocol was constructed and is characterized by a constructivist proposal that aims to provide the main references on the topic as well as using interactive teaching resources to support the teaching-learning process of nursing professionals. However, to verify the effectiveness in learning, it is pertinent that future studies evaluate the applicability of the care protocol, considering the varied use of the material by nurses in the hospital environment, in primary care and in home care.

In this sense, considering the information and image/mental model included in the protocol, nurses will be able to learn new information so that they adopt safe practices and attitudes in their daily lives with a view to rehabilitation of IN. It is concluded, therefore, that the NI rehabilitation protocol is validated and can facilitate the teaching-learning process of nurses in the clinical practice of caring for people with NI.
